# China’s great wall, Israel’s Bar Lev Line, and passive infectious disease surveillance

**DOI:** 10.1186/2054-9369-1-15

**Published:** 2014-07-21

**Authors:** Maha A Elbadry, Mary M Merrill, Meng-Meng Ma, Mai-Juan Ma, Jia-Hai Lu, Wu-Chun Cao, Gregory C Gray

**Affiliations:** One Health Center of Excellence for Research and Training and College of Public Health and Health Professions, University of Florida, P.O. Box 100188, Gainesville, FL 32610 USA; School of Public Health, Key Laboratory of Tropical Disease Control Research, Ministry of Education, Sun Yat-sen University, Guangzhou, Guangdong P.R. China; State Key Laboratory of Pathogen and Biosecurity, Beijing Institute of Microbiology and Epidemiology, Beijing, P.R. China

**Keywords:** Zoonoses, Communicable diseases, Emerging, Epidemiology, Public health, One health

## Abstract

Emerging infectious diseases are some of modern society’s greatest threats. Like some great construction efforts designed to protect mankind, current public health measures against these emerging pathogens have not always been successful. This paper highlights the importance of embracing new interdisciplinary approaches towards emerging pathogen threats. One such approach, termed One Health, is quickly being embraced by professional organizations and public health institutions across the world as a way forward. This paper briefly discusses the above problems and preliminary steps taken by Chinese academic institutions to embrace the One Health approach.

## Main text

Since ancient times, one of the chief reasons people banded together was for the purpose of defending themselves against threats to their safety and well-being. Protective measures have taken many forms over many years. One of the most widely-known marvels of such protection efforts is the Great Wall of China, a sight visited by more than 10 million travelers each year [1].

Historians agree that ~220 B.C. the first Emperor of a newly unified China, Qin Shi Huang, envisioned a grand plan to protect his kingdom from the invading Xiongnu nomadic horsemen of the north. Qin expanded the existing system of defensive walls along the northern border of China into one Great Wall in hopes this barrier would quell the barbarian attacks. Qin succeeded in creating a formidable defense structure, estimated to be 3,100 miles upon completion. The wall included watchtowers built at intervals, with a sophisticated warning and communication system of smoke signals, lanterns, and beacon fires.

Though the Great Wall slowed the attacks, this passive strategy was not enough to end the assaults of the fierce Xiongnu horsemen who often rode in groups of up to 300,000 archers. Roughly 100 years later, Emperor Wudi initiated an active campaign against the barbarians, to finally end their attacks. By sending out strong expeditions to disband the barbarian warrior groups at their sources, the Chinese finally established absolute rule for a period. This historical experience illustrates the need to supplement a passive protective strategy with an effort to actively engage a threat at its source.

In modern history, Israel embraced a similar method for protection by building a 100-mile line of defensive walls and trenches along the eastern side of the Suez Canal to protect Israelis from Egyptian artillery bombardment during the War of Attrition (1967–70). It was estimated that Israel invested $300 million in this massive defensive concrete and sand structure called the Bar Lev Line, reaching a height of 66–82 feet [2]. Just behind the line was the first line of fortification with 22 forts resembling 31 strongpoints. Each strong point was supported with trenches, minefields, barbed wire and up to 26 bunkers armed with medium to heavy machine guns. Israel took almost every possible and potential threat into consideration as they built the line, even considering the installation of an underwater pipe system to pump fuel to ignite the canal and create a sheet of flame if an attack took place. Despite of all these precautionary measures and extensive efforts to keep the enemy outside, the line of Bar Lev was breached in less than two hours, due to the element of surprise and the help of a very simple weapon that was not considered - water. Using a British and German-made water pump in 1973 Egyptians managed to tear down 1,500 cubic meters of sand in two hours creating 81 breaches in the line and allowing troops to pass. Although the line was effective for 4 years, its protective effect did not last when the invaders were determined and their campaign focused.

Similar to the Great Wall Emperor Qin created to protect his nation from invading threats in ancient China, and the Bar Lev line created by Israel in recent times, there exist many “Great Walls of Health” surveillance systems in place to protect the public health of nations today. These systems often depend on surveillance of disease events to alert authorities of the presence of a novel threat and its potential for epidemics. Theoretically, once a novel threat is detected and an alarm resounded, a cascade of events should occur to protect the population. In the case of infectious disease this may include: improved diagnostics, focused surveillance, isolation of infected patients, and various interventions.

While these largely passive surveillance systems are designed to detect and initiate response to novel pathogens, alone they may not be effective enough to stop a threat. A good example is the 2009 H1N1 pandemic where the novel virus spread so quickly across such large geographical areas that nations were unable to fully engage their pandemic response plans [3]. Hence, like a large defensive wall, a passive threat surveillance system alone may not be enough to avert disaster. The challenge today is to combine largely passive emerging disease surveillance systems with modest active engagement programs at the source of a novel pathogen’s generation. We surely need surveillance but we also need an offensive policy similar to the campaigns led by the ambitious Emperor Wudi. In infectious diseases, we need to target potential disease hotspots of novel pathogen generation before novel pathogens fully adapt to their hosts (human or animal) and become more virulent and more efficient at host-to-host transmission.

**Figure 1:**
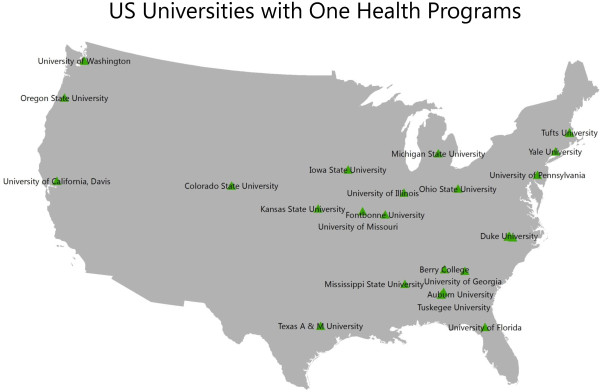
**Map of higher academic institutions in the United States of America currently engaged in One Health-related research or training programs.**

Just as the Great Wall was built to defend in a time when man’s chief military weapons were the sword and arrows, many existing public health strategies were created to defend against disease agents of the past. We must adapt our strategies to match the changing disease landscape. Similar to the breach of the Bar Lev line, novel pathogens can catch us off guard even when we do our best to anticipate them. When we examine today’s infectious diseases, we see that roughly 75% of emerging infectious diseases are zoonotic, meaning they spread between humans and other animals [4]. The study and control of these novel zoonotic pathogens require an integrated, interdisciplinary approach involving human, veterinary, and environmental health experts, also called the “One Health” approach. This approach is being endorsed by many professional and academic organizations including: The World Bank, The US Institute of Medicine, The US CDC, etc. [5–8]. Only through interdisciplinary and often international cooperation can the characteristics and transmission patterns of these novel agents be properly understood and the public health best protected. This concept is being widely embraced internationally. In the US alone at least academic institutions have initiated some sort of One Health research or training programs (Figure 1).

Sun Yat-Sen University, South China Agricultural University, Beijing Institute of Microbiology and Epidemiology, the University of Florida, and multiple Chinese collaborating institutions will soon host an International Symposium for One Health Research, the purpose of which is to foster modern approaches to disease threats. The November 22–23 meetings in Guangzhou China will bring together experts from around the world and across China to examine successful One Health approaches to emerging public health threats such as zoonotic pathogens, food security, antimicrobial resistance, and environmental toxins. More information regarding these meetings can be found at the Symposium web site: http://onehealth.csp.escience.cn/dct/page/1.

## References

[1] **China's Great Wall Crumbles as Tourism Soars** Available at: http://news.discovery.com/earth/great-wall-of-china-deteriorating.htm. Accessed 6/9/2014

[2] **Bar-Lev Line** Available at: http://www.globalsecurity.org/military/world/israel/bar-lev-line.htm

[3] US Department of Health and Human Services (2012). An HHS Retrospective on the 2009 H1N1 Influenza Pandemic to Advance All Hazards Preparedness.

[4] United States Centers for Disease Control and Prevention: **Zoonotic Disease: When Humans and Animals Intersect.** Available at: http://www.cdc.gov/24–7/pdf/zoonotic-disease-factsheet.pdf

[5] Zheng X, Lu J, White SK, Sabo-Attwood T, Gray GC (2014). Adopting and Implementing a One Health Approach for Solving Complex Health Issues in China.

[6] American Medical Association (2007). Resolution: 530. Association AM.

[7] US Centers for Disease Control and Prevention: **Division of High-Consequence Pathogens and Pathology (DHCPP), One Health Office.** Available at: http://www.cdc.gov/ncezid/dhcpp/one_health/index.html. Accessed June 15, 2014

[8] World Organisation for Animal Health (OIE): **Fifth Strategic Plan: 2011–2015.** available at: http://www.rr-africa.oie.int/docspdf/en/2010/5th_strategic_plan.pdf

